# A chimeric antigen receptor against prostate-specific membrane antigen, a tumor vasculature target

**DOI:** 10.1186/2051-1426-1-S1-P33

**Published:** 2013-11-07

**Authors:** Stephen P Santoro, Soorin Kim, Dimitrios Alatzoglou, Daniel J Powell, George Coukos

**Affiliations:** 1 Ovarian Cancer Research Center, University of Pennsylvania, Philadelphia, PA, USA

## 

The tumor endothelium plays a complex role in cancer development. Aberrant blood vessels provide structural support for the tumor, a barrier to immune infiltration, and also serve as a source of pro-tumorigenic signals. In accordance with their atypical function, surface protein expression on tumor vascular cells is distinct. This provides a unique opportunity for adoptive cell therapy. Prostate-specific membrane antigen (PSMA) is highly expressed in the vasculature of a variety of solid tumors, making it an attractive therapeutic target. We therefore designed a series of chimeric antigen receptors (CAR) against PSMA, utilizing an scFv derived from the anti-PSMA antibody, J591. The scFv was linked to each of five intracellular signaling domains: Z, 28Z, BBZ, or 28BBZ. In vitro, we found that all CAR bearing T cells performed equally well against endothelial targets. However, increased induction of the anti-apoptotic protein Bcl-xL in T cells bearing the 28BBZ signaling domain led us to select the 28BBZ CAR for further study (herein known as P28BBZ). IFNγ ELISA and Cr51 release assays confirmed the functionality of the P28BBZ CAR in vitro. To assess the ability of the P28BBZ T cells to recognize PSMA-positive vessels, human endothelial cells were seeded to a Matrigel basement membrane and allowed to form microvessels before co-culture with the CAR bearing T cells. Time-lapse microscopy revealed that the P28BBZ T cells preferentially homed to PSMA-positive vessels, destroying them within 24 hours of co-culture. In vivo, P28BBZ T cells were able to specifically and durably eliminate PSMA-positive hemangiomas. PSMA negative hemagiomas, which were injected on the opposite flank of the same mice, were unaffected by the injection of the P28BBZ T cells. To further mimic the tumor microenvironment, mice were injected on each flank with ID8 ovarian cancer cells mixed with endothelial cells engineered to express a luciferase reporter, or a luciferase reporter with human PSMA. The PSMA positive vessels were specifically eliminated upon administration of the P28BBZ T cells, and a corresponding decrease in tumor volume was observed. Taken together, these data demonstrate that T cells harboring the P28BBZ CAR can specifically eliminate PSMA positive endothelial targets, and that elimination of these cells impairs tumor outgrowth.

